# An optimization framework to guide the choice of thresholds for risk-based cancer screening

**DOI:** 10.1038/s41746-023-00967-9

**Published:** 2023-11-28

**Authors:** Adam R. Brentnall, Emma C. Atakpa, Harry Hill, Ruggiero Santeramo, Celeste Damiani, Jack Cuzick, Giovanni Montana, Stephen W. Duffy

**Affiliations:** 1 Wolfson Institute of Population Health, Queen Mary University of London, London, UK; 2 Sheffield Centre for Health and Related Research, University of Sheffield, Sheffield, UK; 3 Warwick Manufacturing Group, University of Warwick, Coventry, UK; 4 Data Science & Computation Facility, Fondazione Istituto Italiano di Tecnologia, Genoa, Italy

**Keywords:** Risk factors, Cancer screening, Cancer epidemiology, Breast cancer, Statistics

## Abstract

It is uncommon for risk groups defined by statistical or artificial intelligence (AI) models to be chosen by jointly considering model performance and potential interventions available. We develop a framework to rapidly guide choice of risk groups in this manner, and apply it to guide breast cancer screening intervals using an AI model. Linear programming is used to define risk groups that minimize expected advanced cancer incidence subject to resource constraints. In the application risk stratification performance is estimated from a case–control study (2044 cases, 1:1 matching), and other parameters are taken from screening trials and the screening programme in England. Under the model, re-screening in 1 year for the highest 4% AI model risk, in 3 years for the middle 64%, and in 4 years for 32% of the population at lowest risk, was expected to reduce the number of advanced cancers diagnosed by approximately 18 advanced cancers per 1000 diagnosed with triennial screening, for the same average number of screens in the population as triennial screening for all. Sensitivity analyses found the choice of thresholds was robust to model parameters, but the estimated reduction in advanced cancers was not precise and requires further evaluation. Our framework helps define thresholds with the greatest chance of success for reducing the population health burden of cancer when used in risk-adapted screening, which should be further evaluated such as in health-economic modelling based on computer simulation models, and real-world evaluations.

## Introduction

Policy makers design cancer screening programmes by weighing up their benefits from saving lives and reducing morbidity, with costs and risks to participants. All screening tests yield false-positive results, where further investigation of screening abnormalities reveals no cancer. This investigation may involve additional tests including biopsy, causing increased anxiety, and physical discomfort for the patient. On the other hand, not all cancer is detected early by screening, leading to a missed opportunity for early diagnosis and treatment. To increase the effectiveness of current programmes, and to design new screening algorithms, there is increasing interest in risk-adapted cancer screening. Here, screening is tailored to a person’s risk, so that those at highest risk receive the greatest screening activity.

A setting where risk-based screening is being actively considered is breast cancer control^1–4^. A variety of models have been developed to assess the risk of breast cancer. Many use classical risk factors such as family history and reproductive factors, and some include breast density and polygenic risk scores^5^. While most models for breast cancer risk assessment have focused on time horizons from 5 years to lifetime risk irrespective of a screening mammogram result, some have been developed for shorter-term risk after and conditional on a negative screening mammogram result. Recent evidence suggests that information in a woman’s routine screening mammogram is useful in this context and appears to provide more information than classic risk models. Several case–control and cohort studies indicate that this is likely to be because AI models, whether trained to detect cancer at screening or over a longer period of time, can identify subtle early signs of breast cancer on the mammogram^6–9^.

The UK National Health Service (NHS) is exploring the possibilities of using AI and machine learning technologies to help clinicians interpret mammograms in breast screening (https://www.longtermplan.nhs.uk). One system that has been developed for breast cancer risk assessment based on screening mammograms is an AI model called Mirai^10^. Performance of the system in terms of discrimination ability for interval and subsequent screen-detected breast cancers diagnosed over 1–6 years has held up in multiple settings^6–8^. We plan to evaluate AI-guided screening using a model such as Mirai, through a comprehensive health-economic model for risk-based screening to estimate health-related quality of life, cancer survival and NHS costs over the lifetime of the female population eligible for screening in the UK. Evaluation using this model requires defined screening regimens chosen based on risk thresholds. Risk groups for decision making are included in breast cancer clinical guidelines for the longer-horizon risk models based on classical risk factors such as family history, but it is not clear if they are suited for shorter-horizon AI risk models. To help with the choice of thresholds, we therefore aimed to develop a framework to evaluate the potential effectiveness of different thresholds for AI model-based screening strategies. Thresholds identified using this first model may be subsequently evaluated in a more comprehensive health-economic model.

In this paper, we present our framework designed to evaluate the likelihood of advanced cancer based on a risk assessment and screening approach. Its purpose is to help determine the most effective risk thresholds for risk-adapted cancer screening, and also show whether there are potential benefits from risk-stratified screening, and indicate the scale of these potential benefits. In our application, we show how the framework may be applied to assess thresholds for the Mirai breast AI model to tailor the screening interval in those attending the NHS Breast Screening Programme aged 50–70 years.

## Results

### Breast cancer imaging AI

Our framework (see Section ‘Methods’) was applied to risk-adapted mammography screening intervals using an AI algorithm called Mirai. This deep-learning algorithm estimates risk of breast cancer annually to 5 years, using four-view digital mammography^10^. It has been evaluated in a case–control study in women attending the NHS Breast Screening Programme 2010–2019, using mammograms from the OPTIMAM database^8,11^. The AI model was a strong predictor of advanced breast cancer risk at the next screening mammogram.

Our aim is to evaluate the use of this algorithm for risk-stratified screening in an English context. Here, women are currently invited for triennial double-reading mammography screening when aged 50–70 years. In this setting, some studies have considered the health-economic utility of risk stratification to tailor screening, rather than the current one-size-fits-all approach. This includes a life-table analysis^12^ and computer simulation models^13^. Stochastic discrete-event simulation models such as the latter are a powerful and flexible means to evaluate healthcare interventions including risk-based screening. However, they are rarely designed for optimization of parameter inputs such as thresholds, but are intended to provide a rich analysis of the harms and benefits of different risk-based strategies. Our goal is to use the deterministic model in this paper to propose and determine thresholds to use for different screening strategies based on using computer simulation or other such models.

### Model parameters and assumptions

To estimate risk thresholds using the Mirai algorithm, parameters for the optimization model were set as follows, following the notation defined in Methods section.

1. ‘Advanced’ breast cancer was defined to be cancer that has spread to lymph nodes (node-positive disease). Node positivity is one of the strongest factors associated with prognosis in patients diagnosed with breast cancer, and randomized controlled trials have shown that reductions in node-positive cancer are associated with reductions in breast cancer mortality^14,15^. Although other definitions of ‘advanced’ breast cancer might also be used, such as clinical stage 2b or greater, we use node positivity here because data are available from screening trials, and from routine modern screening practice for mammography screening on the proportion of cancers node positive or with missing node status by detection model. In particular, we follow an estimate from ref. ^16^ that 22% screen-detected cancers and 53% interval cancers are node positive (i.e., assumptions for *a*_*kj*_(*t*) and *b*_*kj*_(*t*) as defined in Section Methods). While these might differ by age, the algorithm was only very weakly correlated with age (Spearman correlation 0.18)^8^, and so we judge this a reasonable assumption.
2. Sensitivity of mammographic screening was taken to be *D*_*k*_ = 0.92, taken as constant over all quantiles of risk *k* = 1, …, *n*. The assumption of constancy by quantile of Mirai is justified because an earlier analysis of risk assessment using Mirai found similar discrimination for interval and screen-detected cancers, and the algorithm was only very weakly correlated with the strongest factor associated with sensitivity of mammography: breast density (Spearman correlation 0.15)^8^.
3. The transition rate from asymptomatic to symptomatic disease is *λ*_*k*_ = 0.25 following randomized trial evidence^17^. We do not have any direct evidence on the association between Mirai risk and this parameter, and assume it is constant over all levels of risk.

Based on the above assumptions, model (2) may be used to estimate the proportion of advanced cancers detected by screening interval and mode of detection. The consequence of the above assumptions on advanced cancer risk by screening interval is shown in Table 1.

**Table 1:** Proportion of cancers expected to be node-positive by mammography screening interval following Eq. (1) and assumptions in subsection ‘model parameters and assumptions’.

Interval (years)	Screen detected (%)	Node-positive (%)
1	87	26
2	76	29
3	67	32
4	60	34
5	54	36

**Fig. 1:**
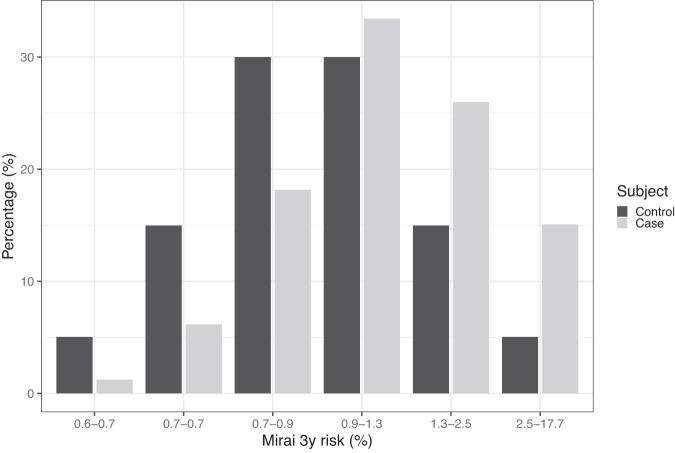
Estimated distribution of 3y risk from the Mirai AI model. The panel shows a histogram of Mirai 3-year risk in breast cancer cases and controls using data from ref. ^8^.

The degree of risk stratification achieved by the AI model over 3 years is shown by the histogram in Fig. 1. Many more women with cancer at the next screen in 3 years are identified in the top 5% of the control distribution compared with the bottom 5%. This means that there is a potential efficacy gain from more intensive screening for those at high which might outweigh the efficacy loss from less intensive screening of those at lower risk.

The distribution of risk in the population was estimated using mean 3-year risk in centiles from the empirical distribution function of the AI model projected 3-year risk in controls. For the optimization problem, we therefore used *K* = 100, with the decision problem to determine *x*_*kj*_, the screening regimen *j* for each centile *k*. Focus is on the relative benefit of risk-based screening compared with triennial screening for all, so that optimization solutions are evaluated by dividing the estimate of advanced cancer incidence *P*(**X**) (Eq. (3)) by the value where everyone receives triennial screening.

The cost functions for strategy *j* do not depend on centile *k* (i.e., *h*_*kj*_ = *h*_*j*_), and are set to be the total number of screens required over a 6-year period; e.g., for biannual screening strategy *h* = 3, for triennial *h* = 2. If the total constraint *H* = 200 then the same number of screens as triennial for all is met; this is also varied to explore results when more or fewer screens (resources) are available.

### Risk groups

To help guide the choice of thresholds, we considered a scenario where time to the next screen is to be determined in 1-, 2-, 3-, 4-, or 6-year intervals. Thresholds were chosen when a subset of these intervals was considered: either (i) 1, 2, 3 or 6 years; (ii) 1, 3, or 6 years; (iii) 2, 3, or 6 years; and (iv) 1, 3, or 4 years. The baseline scenario constrains the total number of screens used to be the same as triennial screening, which is used for all women in the NHS breast screening programme.

The optimal threshold choices using the same resources as triennial screening are shown in Table 2 (see Supplementary Table 1 for corresponding thresholds). When the number of screens was constrained to equal the same as triennial screening on average, then most women were still recommended triennial screening. Allowing four screening intervals (1, 2, 3 and 6 years) reduced the number of advanced cancers diagnosed by approximately 18 advanced cancers per 1000 cancers diagnosed with triennial screening, which was the same as three screening intervals if the low-risk group received 4-year intervals (choices 1, 3 or 4 years). As expected, the other screening strategies were slightly inferior with fewer advanced cancers expected to be prevented, being 16 per 1000 with 1, 3, 6-year options; and 13 per 1000 if 2, 3, or 6-year options. The analysis suggests that if one is operating with constraints on number of screens being as the current English programme, and wishes to offset additional screens needed by a high-risk group from a low-risk group, then one might prefer a slight lag in screening interval for the low-risk group (e.g., 4 years), but a much higher intensity for a relatively small high-risk group (1 year).

**Table 2:** Proportion of the population assigned to different screening intervals following an image AI risk assessment under our model, under a constraint that the average number of screens is the same as the current UK programme (triennial for all).

Possible interval (years)	Interval	Per cent population
2, 3, 6	2	14
	3	72
	6	14
1, 3, 6	1	4
	3	80
	6	16
1, 2, 3, 6	1	3
	2	8
	3	69
	6	20
1, 3, 4	1	4
	3	64
	4	32

**Fig. 2:**
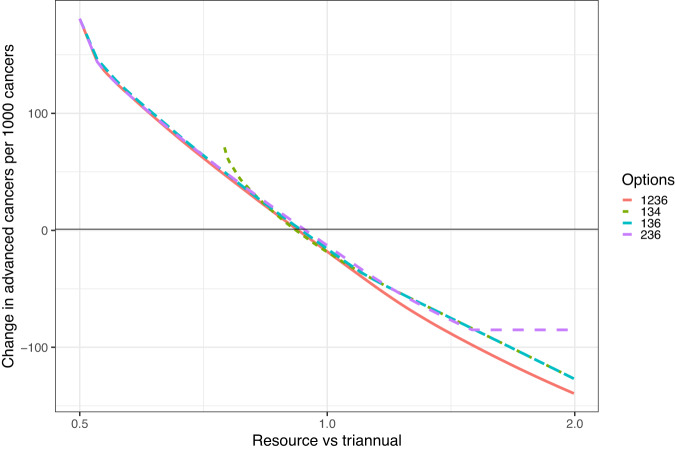
Estimated benefit of risk-adjusted screening as a function of resource. The panel shows change in predicted number of advanced cancers compared with 1000 advanced cancers from triennial screening for all, when the screening intervals available are as shown in Options (e.g., 134 means 1-, 3- or 4-year intervals) as a function of resources relative to triennial for all (*x*-axis = 1.0).

Figure 2 extends this analysis and shows the anticipated advanced cancer incidence from risk-based vs universal triennial screening as a function of resources. It shows that, theoretically, one may obtain greater benefits for the same resource, or achieve the same advanced cancer incidence as triennial screening with less resource. It also shows that having more options for screening interval is more worthwhile when resources increase. For example, the 2-, 3- or 6-year option reaches a maximum when only 2-year screening for all is possible.

**Fig. 3:**
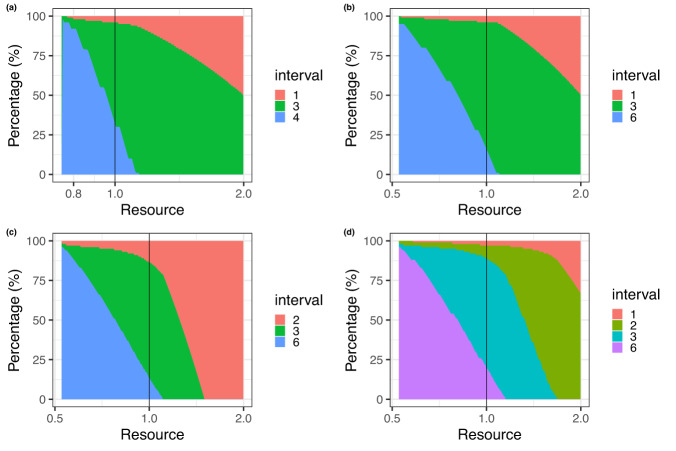
Optimal percentage of the population in risk groups associated with different screening intervals (see legend) as a function of the resources required (number of screens, 1.0 is the same as triennial screening for all), under different screening interval options. **a** is for 1,3, or 4y intervals; (**b**) is for 1,3 or 6y intervals; (**c**) is for 2,3 or 6y intervals; (**d**) is for 1,2,3 or 6y intervals.

Figure 3 plots the percentage population who fall into each screening strategy as a function of resources (total screens). The proportion recommended annual screening is relatively small and stable when resources are slightly increased or decreased relative to triennial screening for all (3% when 2, 3 and 6 years are considered; 4% when only 3 and 6 years are considered). If resources are more constrained than triennial screening, the model suggests one should still prioritize higher-risk subgroups to receive more frequent than triennial screening, rather than say the minimum screening interval in the population to be 3 years.

### Robustness

Estimation of the reduction in advanced cancer risk in the population that would be achieved by risk-stratified screening depends on accurate calibration of all the parameters in the model (*a*_*kj*_, *b*_*kj*_, *r*_*kj*_, *s*_*kj*_ defined in Methods section). However, one useful feature of the optimization model is that choice of risk thresholds does not depend on calibration of absolute risks for *r*_*kj*_(*t*) being correct, provided relative risks are well calibrated. This is due to the form of the objective function and constraints. If the true risk ${\tilde{r}}_{kj}$ say is multiplied by a constant *M*, so that the risk model over-predicts risk if *M* > 1 and underestimates it if *M* < 1, then the optimal solution will not change, as *M* is a scale factor on the objective function and has no effect on constraints. Therefore, to use this model, one simply has to check the calibration of the relative risks of the risk model. Furthermore, if the model is wrong by a scale factor, then the relative benefit seen in Fig. 2 will also be the same. This is because the inaccuracy in absolute risk affects not only the estimate of advanced cancer risk in the risk-stratified approach but also the baseline scenario of triennial screening for all.

The recommended risk stratification thresholds and estimated relative benefit might vary depending on other assumptions. To evaluate the potential sensitivity of findings to these, we conducted some sensitivity analyses. In all of these analyses, we found the optimal thresholds were the same when resources were constrained to be the same as triennial screening. However, the assessment of relative performance could differ. Here we present findings when screening intervals 1, 3 and 4 years were considered because this strategy appeared effective in the first analysis and is relatively simple because it only involves three risk groups.

**Fig. 4:**
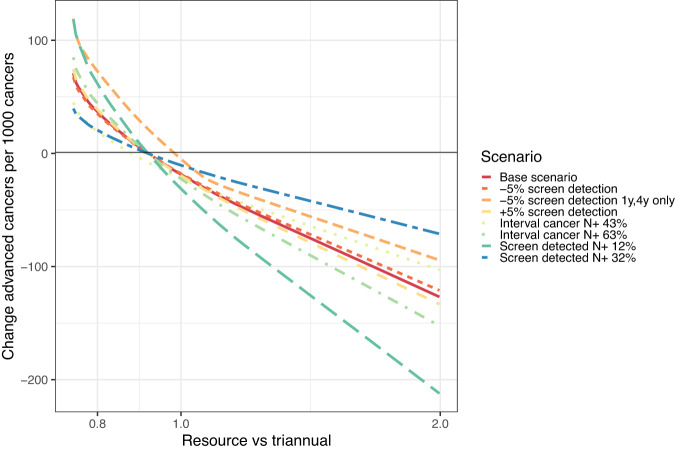
Sensitivity analysis results. The charts shows change in predicted number of advanced cancers compared with 1000 advanced cancers from triennial screening for all when screening intervals 1, 3 or 4 years are available as a function of resources relative to triennial for all (*x*-axis = 1.0), for different sensitivity analyses shown in the legend.

We first varied the assumed node-positive rate by an absolute 10% increase or decrease for screen-detected or interval cancers (*a*_*kj*_ and *b*_*kj*_). We observed a smaller expected benefit when node positivity increased by 10% for screen-detected cancers (reduction of approximately 10 advanced cancers per 1000 with triennial screening), but a greater benefit for the other scenarios (22–31 per thousand). We next varied the assumed proportion screen detected, by increasing or decreasing it by 5%. Estimated benefit was almost unchanged when this change occurred for all groups. Thus, if the sensitivity of the test was lower than anticipated across risk groups, or the rate of disease progression was faster, then we would expect a similar relative benefit compared with triennial screening. We finally assessed when triennial screening would be better than anticipated, with 5% fewer screens detected in the 1 or 4-year regimens only. Here, the anticipated benefit would be much less (approximately 5 per thousand). The effect of these changes in assumptions on relative benefit with varying resources is shown in Fig. 4.

## Discussion

We developed a framework to help choose thresholds for risk-based cancer screening, and applied it to screening intervals following a breast AI risk model. Benefits from screening were modelled to be from the reduction in advanced cancer incidence; costs were constrained indirectly based on the total number of screens in the population. When the total number of screens was constrained to be the same as the current population screening programme as in England, then we identified improvements are theoretically possible by screening those at highest risk annually; offset by reducing the intensity of screening for those at lowest risk. Our results also indicate that there are risk stratification regimens that lead to a reduction in the number of advanced cancers and number of screens. Of the risk-stratified regimens considered, we found using three screening intervals (1, 3 or 4 years) using Mirai 3-year risk thresholds (<1.56% for low risk, and ≥5.06% for high risk) was promising, and expected to reduce the number of advanced cancers diagnosed by approximately 18 advanced cancers per 1000 cancers diagnosed with triennial screening. However, this is an early analysis. Sensitivity analysis suggested that the risk thresholds are likely robust, but the absolute value of the risk-based screening approach was not estimated with precision. The risk thresholds established in this model will feed into a more comprehensive health-economic model to evaluate the net population health benefit of introducing this risk-based screening regimen compared to the current screening programme.

Previous modelling studies suggest that risk-based screening using more static risk factors such as family history and polygenic risk scores might be more cost-effective than the current English screening programme^18^. Largely, results from earlier studies pertain more to who gets screened and at what age, rather than about what the screening regimen is. Even so, there are also practical challenges to this paradigm. For instance, a polygenic risk score requires consent and for a woman to provide DNA for testing. Family history questionnaires must be completed, and IT systems must be put in place for this. If feasible, basing risk assessment on imaging only would eliminate the need for additional data collection, such as genetic, family history and lifestyle information, and seamlessly integrate into existing clinical workflows. Other relevant work on the use of Mirai to guide screening intervals has also been presented using a Markov Decision Process model^19^. This used observed data on cohorts of women attending screening to evaluate new regimens with sequential decision making. Costs were indirectly modelled based on the total number of screens in the population. Benefits from changing screening intervals were taken to arise from the timing of a proposed screen in relation to the observed time of diagnosis in the data, assumed linearly proportional to the time an adaptive interval was to the observed actual cancer detection than the observed screen. That is, if the time of proposed screening prior to the observed diagnosis was closer to diagnosis than the actual previous screen, this would incur a positive reward; if it was further away, it would incur a negative reward. However, it is unclear how this adhoc reward function would relate to advanced cancer incidence or mortality. For example, it takes no account as to whether the observed cancer was screen detected or an interval cancer, or advanced or early stage, as explicitly considered in our framework.

Our approach has several strengths. Firstly, a deterministic model for advanced cancer incidence is mathematically tractable to estimate risk thresholds using an optimization model. Secondly, the framework relies on relatively few inputs (data) and therefore can be readily applied to exploring the potential for health gains from risk-stratified screening for cancers other than breast cancer. Related to this is that the relative simplicity of the methods used to make predictions of the health gains from risk-stratified screening are perhaps more transparent and comprehensible for a decision maker than simulation models. Thirdly, in our view, an attractive feature of the model for threshold choice is it does not require the invasive cancer risk model to be calibrated for absolute risk; only for relative risk. Fourthly, our focus was on screening strategies that maintain the same number of screens as the current programme. This constraint, combined with the risk assessment tool being software applied to screening data already collected at routine appointments, means we expect the cost to the NHS of delivering the risk stratification proposals in this paper to be less than many other proposals for breast screening risk stratification in the research literature^20^. Cost constraint is a current priority for many policy makers in the UK NHS.

Our work has several limitations. Firstly, it does not consider many important aspects of assessing the worth of different screening strategies including harms such as unnecessary biopsies, nor feasibility in terms of implementation. However, the model is only intended to provide preliminary modelling evidence to be evaluated further in more comprehensive health-economic models, or in prospective research studies. Secondly, the model is an idealized scenario, whereby everyone receives the screening interval offered. In practice, one might observe higher or lower compliance depending on the risk assessment, which would affect overall utility. In addition to subsequent attendance, there may also be a potential negative impact of receiving breast cancer risk estimates on women’s worries and attitudes towards breast screening. Research evidence suggests little psychological harm for women and the majority of women in England are interested in having their risk assessed^21^, but this is based on classical models, which are directly interpretable, not image-based AI models, which largely remain a black box as to how they work and require further investigation. Thirdly, our focus on screening strategies that maintain the same number of screens as the current programme is unlikely to correspond directly with a cost-effectiveness analysis. However, health systems typically aim to implement the most cost-effective option providing there is funding for it. In addition, we do not analyse costs and hence do not ascertain ‘cost-effectiveness’ and we do not consider that programmes might increase the number of screens; it might be that triennial screening the low-risk group is cost-effective despite it increasing the number of screens beyond the current screening programme. Fourthly, we stratified based on relative risk thresholds that do not vary by age, but there is other modelling evidence suggesting that it may be preferable to introduce risk thresholds that vary by age^22^. This was done in our example in order to evaluate a method for tailoring screening interval when all women 50–70 years are offered it. However, the methodology could also be adapted for alternative age-stratified risk-based screening strategies. Finally, our work does not address many important issues for implementation. For example, extending the screening interval for some might be unacceptable to the population. A qualitative UK study reported that many women ‘low-risk’ from classical models ‘did not believe screening should stop altogether’^23^. For any change to a successful screening programme, there must be an understanding of these issues, clear communication on reasons for change and potential benefits and risks from participation.

In conclusion, we developed and applied an optimization algorithm to help evaluate risk-based screening thresholds. It may be used to establish when risk models are good enough for health gains to be theoretically possible from risk-stratified screening compared to the current screening programmes. The framework could be used for other breast cancer risk models by using estimates of their distribution of risk in the population. It could be applied to other cancer types by adapting the model for advanced cancer incidence. It is intended as a first step to help define risk-based strategies that have the greatest chance of success on reducing the population health burden of cancer, that would be considered further in health-economic modelling and real-world evaluations.

## Methods

Our method aims to improve population health by choosing cancer screening intervals based on invasive cancer risk assessment. Risk groups are chosen to minimize an estimate of advanced cancer risk in the population, subject to resource constraints. Minimization of expected advanced cancer risk is likely to be a worthwhile objective for several cancer types. Some cancer screening trials have shown that the ability to shift detection of ‘advanced’ cancers to an early stage where treatment is more effective is linked to the effectiveness of the test to reduce cancer-specific mortality^24^. New screening strategies that reduce ‘advanced’ cancer risk are likely to further improve outcomes over the population. Indeed, this concept is used to justify primary endpoints for large breast cancer screening trials such as the ongoing Tomosynthesis Mammographic Imaging Screening Trial (TMIST)^25^. However, the decision on the definition of ‘advanced’ cancer for the modelling will vary according to cancer type, associated prognosis, and data available for model parameters.

We next outline the method when determining screening regimens for a cohort, then the model is adapted to decide thresholds for a population.

### Screening regimens in a cohort

#### Advanced cancer risk

We first describe our strategy to evaluate expected advanced cancer risk conditional on *Z* risk individual factors **z**_*i*_ = (*z*_*i*1_, …, *z*_*iZ*_), and possible screening regimens *j* = 1, …, *m*, in a cohort of *i* = 1, …, *n* individuals. Let *T* denote a random variable for time to invasive breast cancer diagnosis, given the person has not previously had cancer. The aim is to evaluate advanced cancer risk conditional on the risk factors and screening regimen:

$${p}_{ij}(t)=P(T\le t,\,{{\mbox{stage}}}={{\mbox{advanced}}}\,|\, {{{{\bf{z}}}}}_{i},\,{{\mbox{screening regimen}}}\,=j).$$

This will be used to choose regimens for each individual *i* through an optimization model. We assume that a model for (conditional) invasive cancer risk is available:

$${r}_{ij}(t)=P(T\le t\,|\, {{{{\bf{z}}}}}_{i},\,{{\mbox{screening regimen}}}\,=j).$$

In many settings, invasive cancer risk will be independent of a screening strategy with sufficient follow-up time. One might choose from a range of cancer risk models that have been developed for this. To extend the invasive cancer model to advanced cancer risk, we note prognosis usually differs substantially between cancer (1) detected at screening, or (2) in the interval between screens. We define the chance of screen-detected (vs interval) cancer conditional on a diagnosis of cancer by time *t*, risk factors **z**_*i*_, and screening regimen *j* as

$${s}_{ij}(t)=P(\,{{\mbox{detection}}}={{\mbox{screen}}}\,| T\le t,{{{{\bf{z}}}}}_{i},\,{{\mbox{screening regimen}}}\,=j),$$

so that 1 − *s*_*ij*_(*t*) = *P*(detection = interval∣*T* ≤ *t*, **z**_*i*_, screening regimen = *j*). For most cancers, a model will be needed to estimate *s*_*ij*_(*t*). We propose an epidemiological model based on the transition from asymptomatic to symptomatic disease, taken as constant over the follow-up time *t*. Let the transition rate per year from asymptomatic to symptomatic disease conditional on risk factors **z**_*i*_ and screening strategy *j* be denoted *λ*_*ij*_. The screening interval for individual *i* strategy *j* is *u*_*j*_ years, taken as the same for all *i* given *j*; and test sensitivity of regimen *j* for individual *i* is denoted *D*_*ij*_. Then, following Launoy et al.^26^, we have that approximately:

$${s}_{ij}(t)=\frac{{D}_{ij}\{1-\exp (-{\lambda }_{ij}{u}_{j})\}}{{\lambda }_{ij}{u}_{j}\{1-(1-{D}_{ij})\exp \left.\right(-{\lambda }_{ij}{u}_{j}\}}.$$

To use this for advanced cancer risk, we further define

$${a}_{ij}(t)=P(\,{{\mbox{stage}}}={{\mbox{advanced}}}\,| \,T\le t,{{\mbox{detection}}}={{\mbox{screen}}}\,,{{{{\bf{z}}}}}_{i},\,{{\mbox{screening regimen}}}\,=j)$$

so that 1 − *a*_*ij*_(*t*) = *P*(stage = early ∣ *T* ≤ *t*, detection = screen, **z**_*i*_, screening regimen = *j*). Let the same for interval cancer be denoted

$${b}_{ij}(t)=P(\,{{\mbox{stage}}}={{\mbox{advanced}}}\,|\, T\le t,{{\mbox{detection}}}={{\mbox{interval}}}\,,{{{{\bf{z}}}}}_{i},\,{{\mbox{screening regimen}}}\,=j).$$

Then,

1 $$P(\,{{\mbox{stage}}}={{\mbox{advanced}}}\,|\, T\le t,{{{{\bf{z}}}}}_{i},\,{{\mbox{screening regimen}}}\,=j)={a}_{ij}(t){s}_{ij}(t)+{b}_{ij}(t)\{1-{s}_{ij}(t)\}$$

and so

2 $${p}_{ij}(t)={r}_{ij}(t)[{a}_{ij}(t){s}_{ij}(t)+{b}_{ij}(t)\{1-{s}_{ij}(t)\}].$$

One interpretation of formula (2) is that the model for invasive cancer *r*_*ij*_(*t*) is extended to advanced cancer risk through an adjustment factor. The latter reflects the effectiveness of the screening regimen *j* in reducing risk of advanced cancer, linked to screening test sensitivity, the rate of disease progression, test sensitivity, and screening frequency.

#### Optimization

The conditional model of advanced cancer risk may be used to estimate the best screening regimen *j* = 1, …, *m* for individual *i* = 1, …, *n* given constraints on resources through the following optimization model.

Denote the binary decision variable *x*_*ij*_, where *x*_*ij*_ = 1 if screening regimen *j* is chosen for individual *i*, and *x*_*ij*_ = 0 if not. Our objective is to minimize the expected advanced cancer detection rate

$$\mathop{\min }\limits_{{{{\bf{X}}}}}\mathop{\sum }\limits_{i=1}^{n}\mathop{\sum }\limits_{j=1}^{m}{x}_{ij}{p}_{ij}(t)$$

for the strategy defined by the *n* × *m* matrix **X**.

The constraints are:

1. The decision variable is binary: *x*_*ij*_ ∈ (0, 1) for all *i* = 1, …, *n*; *j* = 1, …, *m*.
2. One screening regimen per person: ∑_*j*_*x*_*ij*_ = 1 for *i* = 1, …, *n*.
3. Resources are constrained: ∑_*i*_∑_*j*_*h*_*ij*_*x*_*ij*_ ≤ *H* where *h*_*ij*_ is the cost associated with screening strategy *j* for individual *i*, and *H* is the total cost. For instance, *h*_*ij*_ might be the number of screens done, and this is constrained to a fixed total number *H*.

Technically, this form of optimization problem is called an integer programme, and may be solved using standard algorithms^27^.

### Choosing risk thresholds for a population

The above optimization model can be used to determine optimal screening strategies on an individual basis, provided all parameters are known or estimable. We next adapt the methodology for population stratification by making the following assumptions.

1. The distribution function of invasive cancer risk in the target population is known. We use the index *k* = 1, …, *K* to denote quantiles, for instance, using centiles, we have *K* = 100. Then, for simplicity, we use the same notation as the previous section but replace index *i* with *k*. The conditional expected invasive cancer risk in each quantile *k* is
  $$\begin{array}{rcl}{r}_{kj}(t)&=&P(T\le t\,| \,{{\mbox{quantile}}}=k,{{\mbox{screening regimen}}}\,=j).\end{array}$$
2. *r*_*kj*_(*t*) = *r*_*k*_(*t*). In other words, average risk in quantile *k* is the same irrespective of screening. This is likely to be true or approximately true for many screening tests. For example, it is expected to hold for mammography screening for breast cancer. An example where it is unlikely to hold is for screening tests that prevent cancer through the detection and subsequent treatment of precursor lesions. For example, human papillomavirus testing for cervical cancer screening.
3. Sensitivity *D*_*kj*_ = *D*_*j*_ does not depend on risk quantile *k*. This assumption is met if the risk factors **z**_*i*_ used are not associated with the performance of the screening test. If this is unlikely, then a stratified estimate might be considered, where risk thresholds used are taken to depend on levels of **z** associated with test sensitivity. In other words, risk stratification depends both on the risk score, and a factor used in the risk score that is also associated with test sensitivity. One example would be a breast cancer risk model that includes mammographic density, which is both a strong risk factor and hinders the test due to masking effects. If the same screening test is used, but, e.g., only the screening interval is varied between regimens *j* = 1, …, *m*, then this may simplify further to *D*_*j*_ = *D*.
4. More effective strategies cost more. That is, resources are directly associated with advanced cancer risk, so that regimens with higher costs also yield greater improvements to advanced cancer risk: *h*_*kj*_(*t*) ≥ *h*_(*k*−1)*j*_ for *k* = 1, …, *K* − 1 and *j* = 1, …, *m*.

Under these assumptions, the integer programme above may be adapted as follows:

#### Objective

Define

3 $$P({{{\bf{X}}}})=\mathop{\sum }\limits_{k=1}^{K}\mathop{\sum }\limits_{j=1}^{m}{x}_{kj}{p}_{kj}(t)$$

for strategy defined by the *K* × *m* matrix **X**, where

$${p}_{kj}(t)=[{a}_{kj}(t){s}_{kj}(t)+{b}_{kj}(t)\{1-{s}_{kj}(t)\}]{r}_{k}(t).$$

The objective is

$$\mathop{\min }\limits_{{{{\bf{X}}}}}P({{{\bf{X}}}}).$$

#### Constraints

1. The decision variable ${x}_{kj}\in {\mathbb{R}}$ and 0 ≤ *x*_*kj*_ ≤ 1 for all *k* = 1, …, *K* and *j* = 1, …, *m*.
2. Everyone in a quantile is assigned a screening strategy: ∑_*j*_*x*_*kj*_ = 1 for *k* = 1, …, *K*.
3. Resources are constrained: ∑_*k*_∑_*j*_*h*_*kj*_*x*_*kj*_ ≤ *H* where *h*_*kj*_ is the cost associated with screening strategy *j* for quantile *k*, and *H* is the total cost.

The linear programme formulation is less computationally expensive to solve than an integer programme, and the optimal solution may place thresholds part way within risk quantiles.

### Data

In our application, we estimate the distribution of invasive cancer risk by quantile of Mirai in the population of women attending the NHS breast screening programme; we use data from a case–control study that was designed to evaluate the AI model. These are *n* = 2044 cases matched 1:1 to controls on age, mammography equipment, and site, aged 47–70 years (median 60 years, IQR 55–65 years) attending one of two sites between 2010 and 2019^8^. The source data used in this study are available from the OPTIMAM registry (https://medphys.royalsurrey.nhs.uk/omidb/getting-access/). These data were fully anonymized and received ethical approval for research (research ethics committee (REC) reference: 19/SC/0284, IRAS reference: 265403).

### Reporting summary

Further information on research design is available in the Nature Research Reporting Summary linked to this article.

### Supplementary information

Supplementary Material

Reporting Summary

## Data Availability

The source data used to generate further data for this study are available through the application to the OPTIMAM Mammography Image Database, with further information via this link https://medphys.royalsurrey.nhs.uk/omidb/getting-access/. All data used to construct the tables and figures in this paper are available in GitHub.com via this link https://github.com/brentnall/risk-based-screening-groups.

## Code availability

All code used in this study for analysis is available on GitHub.com and can be accessed via this link https://github.com/brentnall/risk-based-screening-groups. The Mirai algorithm and source code are available on GitHub.com and can be accessed via this link https://github.com/yala/Mirai.
