# Supplementary material for: An optimization framework to guide the choice of thresholds for risk-based cancer screening
Source: NPJ Digit Med. 2023 Nov 28;6:223. doi: 10.1038/s41746-023-00967-9 (PMC10684532; doi:10.1038/s41746-023-00967-9)
Supplement: Supplementary file 1 — Supplementary Material [file 41746_2023_967_MOESM1_ESM.pdf]

Title: An optimization framework to guide choice of thresholds for risk-based cancer screening: Supplementary Material

Authors: Adam R Brentnall, Emma C Atakpa, Harry Hill, Ruggiero Santeramo, Celeste Damiani, Jack Cuzick, Giovanni Montana, Stephen W Duffy

Supplementary Table 1 Mirai 3y risk cutpoints for centiles of the sample, and average predicted risk within each centile.

Centile	Lower cutpoint, 3y risk (%)	Average 3y risk (%)
1	1.18	1.20
2	1.21	1.21
3	1.22	1.23
4	1.23	1.24
5	1.25	1.25
6	1.26	1.26
7	1.27	1.28
8	1.29	1.29
9	1.30	1.30
10	1.31	1.31
11	1.32	1.32
12	1.33	1.33
13	1.34	1.34
14	1.35	1.35
15	1.36	1.37
16	1.37	1.37
17	1.38	1.38
18	1.39	1.39
19	1.40	1.41
20	1.41	1.42
21	1.42	1.43
22	1.43	1.44
23	1.44	1.45
24	1.45	1.46
25	1.46	1.47
26	1.47	1.48
27	1.48	1.49
28	1.49	1.50
29	1.51	1.51
30	1.52	1.52
31	1.53	1.54
32	1.54	1.55
33	1.56	1.57
34	1.57	1.58
35	1.58	1.59
36	1.60	1.60
37	1.61	1.62
38	1.62	1.63
39	1.64	1.64
40	1.65	1.66
41	1.66	1.67
42	1.69	1.69
43	1.70	1.71
44	1.71	1.72
45	1.73	1.73
46	1.74	1.75
47	1.76	1.77
48	1.79	1.80
49	1.81	1.82
50	1.82	1.83
51	1.84	1.85
52	1.86	1.87
53	1.88	1.88
54	1.89	1.90
55	1.91	1.92
56	1.93	1.95
57	1.96	1.97
58	1.98	1.99
59	2.00	2.01
60	2.01	2.03
61	2.04	2.05
62	2.06	2.07
63	2.09	2.10
64	2.10	2.11
65	2.12	2.14
66	2.15	2.16
67	2.17	2.18
68	2.20	2.21
69	2.23	2.24
70	2.24	2.26
71	2.27	2.28
72	2.30	2.32
73	2.34	2.36
74	2.37	2.39
75	2.40	2.41
76	2.42	2.44
77	2.46	2.47
78	2.49	2.51
79	2.52	2.54
80	2.57	2.59
81	2.61	2.62
82	2.64	2.66
83	2.68	2.70
84	2.72	2.73
85	2.75	2.77
86	2.79	2.82
87	2.86	2.89
88	2.92	2.95
89	2.98	3.00
90	3.03	3.07
91	3.10	3.14
92	3.18	3.25
93	3.34	3.45
94	3.55	3.64
95	3.77	3.96
96	4.27	4.62
97	5.06	5.79
98	6.59	7.76
99	8.98	10.01
100	11.49	15.66
